# Maternal and Fetal Outcomes after Multiple Cesarean Deliveries

**DOI:** 10.3390/jcm13154425

**Published:** 2024-07-29

**Authors:** Laura E. Muñoz-Saá, Rebeca Sendra, Isabel Carriles, Mafalda Sousa, Miriam Turiel, Álvaro Ruiz-Zambrana, Luis Chiva

**Affiliations:** Department of Obstetrics and Gynaecology, Clínica Universidad de Navarra, Marquesado de Sta. Marta St, 1, 28027 Madrid, Spain

**Keywords:** multiple repeat cesarean delivery, pregnancy outcome, maternal morbidity, neonatal morbidity

## Abstract

**Background/Objectives:** Cesarean delivery (CD) is a common procedure, but it can be associated with some increasing risks as the number of previous CD increases. Although women undergoing multiple CDs is very unusual in Spain, our center serves pregnant women with a history of three or more previous CDs with some frequency. We aimed to assess whether women who undergo multiple CDs (≥4) have more risks than those who undergo a third CD. **Material and Methods:** A retrospective cohort study was conducted with 161 pregnant women who had undergone ≥ 2 previous CDs and were monitored during their next pregnancy. The primary endpoint was to evaluate the obstetric hemorrhage rate in the multiple CD group and compare it with that in the third CD group. Secondary outcomes regarding maternal and neonatal complications were also analyzed. **Results:** Hemorrhage (7% and 10%; *p* = 0.522) and transfusion (3% and 8%; *p* = 0.141) rates were similar in both groups. The risk of dehiscence of the uterine segment (6% and 24%; *p* < 0.006), as well as hysterectomy (0 and 6.6%, *p* = 0.019), difficult abdominal opening (49% and 82%; *p* = 0.001), peritoneal adhesions (3% and 22%; *p* < 0.001), and difficult bladder separation (36% and 73%; *p* < 0.001), was higher in the multiple CD group. No uterine rupture or maternal-neonatal mortality was observed in either of the groups. **Conclusions:** Since undergoing multiple CD is uncommon, our study may be the largest sample in our environment. Our findings suggest that despite the potential risks of undergoing multiple CDs, maternal and neonatal outcomes are overall favorable.

## 1. Introduction

Cesarean delivery (CD) is currently a safe and common obstetric procedure [1]. However, CD rates have increased alarmingly in recent decades in Western countries. In the USA, the CD rate increased from 5.5% in 1970 to ~32% in 2020 [1,2]. In Spain, cesarean deliveries represented approximately 25% of all births in 2021 [3]. These figures are much higher than those recommended by medical societies and the World Health Organization (WHO), which set the ideal rate at approximately 10–15% [4]. Although medical advances and improvements in surgical and anesthetic techniques and other advances in medicine, such as the use of antibiotics or the availability of blood banks, have made CD a safe procedure, no evidence supports benefits for women or infants when the procedure is not medically justified [1,4]. Indeed, CD can be associated with short- and long-term risks, higher than associated with vaginal delivery [1,2]. 

Therefore, the number of women with a history of cesarean sections has also increased. The existence of one or more previous cesarean sections conditions the route of the next delivery, promoting repeated cesarean sections, and increasing the risk of placental abnormalities, such as placenta previa and placental accretism. It is also the main risk factor for obstetric emergencies such as postpartum haemorrhage, peripartum hysterectomy, and uterine rupture. Other consequences include abdominal and pelvic adhesions, venous thromboembolism, and associated long-term complications such as chronic pelvic pain and future infertility or subfertility [1,2]. 

Given the fact that the main objective of the different scientific societies in recent years has been to avoid unnecessary caesarean sections, and that the primary strategy has been to avoid the first caesarean section and to attempt vaginal delivery in women with a previous caesarean section, we now find a significant number of women facing the reality of repeated or iterative caesarean section. Various studies have been carried out to assess the risks and delivery routes in women with one and two previous caesarean sections, to consider the risks of third or more caesarean sections, to quantify the increase in risk according to the number of caesarean section orders, and to try to determine a safe threshold above which a new gestation could involve risks that are difficult to assume [1,5].

Iterative or repeat caesarean sections are those that are electively indicated because of a history of one or more previous caesarean sections. For years it has been considered that having a previous caesarean section implied a subsequent birth by caesarean section, creating a vicious circle in which the pregnant woman was already condemned to have all her children by caesarean section, taking into account the associated risks, or to limit her offspring. Appropriate medical advice to pregnant women with a history of caesarean sections must take into account the risks of multiple caesarean sections, in addition to the risk of labour in an operated uterus and the remote possibility of uterine rupture [1,6]. 

In view of the risks described above, bilateral tubal ligation is a recommended practice in our setting in the context of a third caesarean section. Only in certain socio-cultural contexts, and more frequently in other distant geographical areas, women choose a new pregnancy and a fourth caesarean section. It is not uncommon for women who, for different reasons, reject sterilization in Spain to come to our centre to monitor their pregnancy and care for their new multiple caesarean section. This circumstance, which is common in other non-European geographical regions (Brazil, Saudi Arabia, Pakistan, etc.), is not frequent in Spain or in Europe [7].

The growing number of CDs leads to an increasing number of women who undergo repeat CD, in part because a history of previous CD conditions the selection of the following type of delivery [1,8]. With some frequency, in women with previous CDs, elective CD is performed to avoid the risks of labor without considering the potential risks of repeat CDs, such as placental abnormalities and obstetric emergencies [9]. Various studies have attempted to assess the risks of repeat CD and the most adequate delivery route for these women [9,10]. They have also aimed to determine the increasing risk depending on the number of previous CDss to establish a safe threshold beyond which a new gestation could pose risks that are difficult to assume [8,9]. According to a systematic review, maternal morbidity progressively increased as the number of previous CDs increased [9]. In particular, the rates of hysterectomy, blood transfusions, adhesions, and surgical injuries increased with the number of CDs. The incidence of some placental abnormalities (e.g., placenta previa or placenta accreta spectrum (PAS)) was also higher in women with multiple CDs [9].

Trial of labor after cesarean (TOLAC) is recommended for women with one previous CD to reduce potential complications, and repeat CD is normally suggested in women with ≥2 previous CDs [8]. However, vaginal birth may be proposed for some women with two previous CDs, always considering the higher risk of uterine rupture [8]. Bilateral tubal ligation is commonly recommended in the context of a third CD due to the risk of uterine rupture and other maternal complications. However, in specific sociocultural contexts, women reject sterilization, opting for a new pregnancy and CD [6,10,11]. Although it is uncommon for women in Spain and other European countries to refuse sterilization in these circumstances, in our center, it is frequent to treat women with a previous CD who choose a new pregnancy and a multiple CD.

Therefore, our study aimed to evaluate the increased potential risk of multiple CDs (four or more CDs) in tertiary obstetric care center in Spain compared to that of a third CD. In particular, we analyzed whether women undergoing multiple CDs have a higher risk of obstetric hemorrhage than those undergoing a third CD.

## 2. Materials and Methods

### 2.1. Study Design

A retrospective cohort study was carried out with pregnant women who presented to one of the headquarters of the tertiary health care center Clínica Universidad de Navarra, located in Madrid and Pamplona (Madrid and Pamplona, Spain), from 1 January 2010 to 31 March 2022 for pregnancy monitoring. Both headquarters have coordinated obstetric and surgical protocols and professional teams.

The inclusion criteria were pregnant women with a single live fetus and at least two previous CDs. The exclusion criteria were twin pregnancies, fetuses with malformations, previous uterine surgery with cavity entry other than a low transverse incision, HIV infection, and pregnant women with two previous CDs who actively requested to attempt a vaginal delivery. 

All included women signed written informed consent forms, and the study was approved by the Ethics Research Committee with the number 2023.058.

A database was generated after the women’s medical records were reviewed by the medical team that attended to the patients and had legitimate access to their personal data. The database was pseudonymized prior to it being sent to investigators to comply with Organic Law 3/2018 on the protection of personal data and guarantee of digital rights.

### 2.2. Endpoints and Variables

The primary endpoint was the obstetric hemorrhage rate in women who underwent multiple CDs (≥4) compared with those who underwent a third CD. The secondary endpoint was the potential risk of other maternal and neonatal complications in women undergoing a third or a multiple CD.

Data on maternal sociodemographic and gestational characteristics, maternal complications, information related to the surgery and the postoperative period, and neonatal data were collected. The maternal complications considered were postpartum hemorrhage and its severity, transfusion requirement, peripartum hysterectomy, uterine rupture, admission to the intensive care unit (ICU), placenta previa, placenta accreta, bladder and bowel injury, and other complications (wound seroma, thrombosed hemorrhoid, endometrioma in the scar, postoperative pneumonia, arterial hypertension during the postpartum period, and postdural puncture headache). The analyzed data from the neonates were weight, arterial pH, Apgar score at 1 and 5 min, and neonatal intensive care unit (NICU) admission and the reason for admission.

### 2.3. Data Collection and Pregnancy Follow-Up

Pregnancy monitoring followed the center protocol for pregnant women with two or more previous CDs and was performed every month. During the first visit, maternal epidemiological and obstetric data were collected, including the date and type of the previous CD and gestational age. The first-trimester visit included an ultrasound evaluation to rule out cesarean scar gestation. Considering that a previous CD is a risk factor for placenta previa and placenta accreta, during the second and third trimesters, routine ultrasounds were also used to assess uterine segment and placental location. If these complications were suspected, high-resolution ultrasound and/or intense monitoring were recommended.

All patients were informed about the delivery route in the first and third trimesters. Following the protocol, pregnant women with two previous CDs underwent a third elective cesarean delivery at 39 weeks. All women who had three or more previous CDs underwent an elective cesarean delivery between 38 and 39 weeks. The surgery was performed by a senior surgical team, using the Pfannenstiel incision and low transverse segmental cesarean section for the opening in most cases, and a two-layer hysterorrhaphy for the closure in all cases. 

### 2.4. Sample Size Calculation and Statistical Analysis

For the sample size calculation, similar studies were reviewed. Hemorrhage was reported in 3% of women undergoing a third CD and in 8% of those undergoing a fourth or more CD. Assuming this increase and a statistical power of 80% with a confidence level of 95% and a loss rate of 5%, the required sample size was 342 patients for each study group.

Continuous variables are expressed as the mean and standard deviation (SD), and categorical variables are presented as absolute and relative frequencies. Student’s *t* test, the nonparametric equivalent Mann-Whitney U test, and the median distribution test were used for normally distributed quantitative variables. In all cases, the distribution of the variable was verified against the theoretical models, and the hypothesis of homogeneity of variances was contrasted. Chi-square and Fisher’s tests were performed for qualitative variables. Statistical significance was established at *p* < 0.05. The software package used for the analysis was SPSS for Windows Version 27.0.

## 3. Results

**Figure 1:**
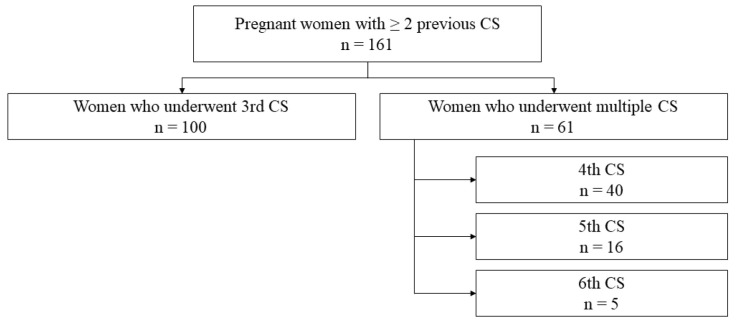
Flowchart.

A total of 161 women with ≥ 2 previous CDs were included in the study. Of these women, 100 underwent a third CD and 61 underwent multiple CDs (four or more CDs). Of the multiple cesarean section group, 40 were fourth, 16 were fifth, and 5 were sixth cesarean sections. Therefore, in the multiple CD group, most patients were undergoing a fourth CD (Figure 1). No differences were found in sociodemographic and gestational characteristics between the groups (Table 1).

More women underwent a scheduled cesarean delivery in the multiple CD group, but the difference was not statistically significant (91.8% vs. 86.0%, *p* = 0.268; Table 2). The mean gestational age exceeded 37 weeks in both groups but was significantly lower in the multiple CD group (*p* = 0.034). Low transverse incision was the predominantly performed surgery in both groups (Table 2). Statistically significant differences were found in the condition of the uterine segment (*p* = 0.006). In particular, dehiscence was more frequent in the multiple CD group than in the third CD group (24 vs. 6%). Opening the abdominal wall and separating the vesicouterine plica was more difficult in the multiple CD group (*p* < 0.001 for both). Peritoneal adhesions were also more frequent in the multiple CD group (*p* = 0.001), and the surgeries were significantly longer (76 ± 32 vs. 62 ± 19 min, *p* = 0.001; Table 2).

A low proportion of women had postpartum hemorrhage, which was resolved with drugs in most cases (Table 3). The drugs used in postpartum hemorrhage were uterotonics, in the following order: oxytocin, methylergometrine, carboprost, and misoprostol, as well as tranexamic acid, according to the center’s protocol. No differences in the hemorrhage rates or hemorrhage severity were found (*p* = 0.522 and *p* = 0.808, respectively). Similarly, the proportions of women who needed a blood transfusion and were admitted to the ICU were low, and similar in both groups (Table 3). Only four women from the multiple CD group needed a peripartum hysterectomy. Three of them were undergoing their sixth CD, and uterus reconstruction was not possible. The other woman underwent a hysterectomy after her fourth CD because of evidence of placental accreta with massive bleeding that required ICU admission and a blood transfusion. Furthermore, there were two bladder injuries in the multiple CD group; one was in a woman undergoing her fourth CD, and the bladder wall was reinforced without opening it. The other was a ureteral injury in the context of the fourth CD with placenta accreta and massive bleeding described above, with venous thromboembolism as a final added complication. The evolution of this case was favorable (Table 3). Placenta previa was diagnosed in two women who were undergoing their third and fourth CD. There were no surgery-related bowel injuries. Other complications are described in Table 3, with no differences between the study groups.

No evidence of uterine rupture was observed among the included women, and there were no cases of maternal or neonatal mortality in the sample. No differences between the groups were found in the neonatal variables analyzed (Table 4).

## 4. Discussion

Several studies have reported a progressive increase in maternal morbidity with an increasing number of previous CDs [9]. However, with the currently available information, it is difficult to establish a safety threshold or a maximum number of CDs above which the risk would not be acceptable. In general, our study showed that women undergoing multiple CDs did not present higher rates of hemorrhage or other maternal complications than women undergoing a third CD, except for the incidence of obstetric hysterectomy.

Some studies have reported a higher risk of postpartum hemorrhage, blood transfusion, and admission to the ICU in patients with multiple cesarean deliveries. Narava et al. (2020), in a retrospective study with a large sample of women with ≥1 CD (*n* = 1008), described an increased mean blood loss with an increasing number of CDs (bleeding of >2000 mL was observed in 1.3%, 1.7%, 4.3%, and 14.3% of the women undergoing a third, fourth, fifth, and sixth CD, respectively; *p* < 0.001), as well as increasing blood transfusion rates (from 2.3% and 2.4% in women undergoing a third or fourth CD to 5.8% and 14.3% for those undergoing a fifth or sixth CD; *p* < 0.001) [12]. However, studies with a lower sample size, comparable to ours, obtained similar results to those presented herein. Uyanikoglu et al. (2016) did not find differences in the incidence of peripartum hemorrhage and blood transfusion in women with four or more CDs compared to those with three or more [10]. In a previous study, Juntunen et al. (2004) did not observe differences among women with multiple CDs (4th to 10th CD) regarding postpartum hemorrhage (*p* = 0.71) [13]. The four peripartum hysterectomies reported in our study were performed in the multiple cesarean section group (0% versus 6.6%, *p* = 0.019), three of which were performed due to the impossibility of reconstructing a polyoperated uterus and one was due to massive bleeding in a pregnant woman diagnosed with placental accreta. The interesting meta-analysis by Akker from 2016 exposes at length the global situation of peripartum hysterectomy across the world. Topping the list of indications are abnormal placentation (38%), uterine atony (27%), and uterine rupture (26%). The risk of hysterectomy is multiplied by 11 if the method of termination of pregnancy under study is a cesarean section (11.38, CI 9.28–13.97) and by 7.5 (OR 7.5, 95% CI, 5.1–11.0) if the patient has a history of a previous cesarean section [14]. Regarding multiple cesarean sections, in the previously mentioned work by Narava et al., they present 1% of obstetric hysterectomy in third cesarean sections, no cases in the group of fourth and fifth cesarean sections, and 10% in the group of sixth cesarean sections [12]. It is worth pointing out the prospective and multicenter study by Silver in which he also found a higher rate of peripartum hysterectomy for higher-order cesareans (0.9% for third cesareans versus 3.4% and 8.9% for the fifth and sixth or more cesarean sections respectively) [15].

Furthermore, published studies coincide with ours on the more significant difficulty in performing surgery for women undergoing multiple CDs. A tendency toward more adhesions with more previous CD has been described [6,16,17,18]. In fact, adhesions seem to be more common and denser after a third CD. Alshehri et al. (2019) reported adhesions as the most frequent complication in the group with multiple CDs compared to the group with low-order CDs [17]. Morales et al. (2007) also reported a higher incidence of adhesions in women with multiple CDs, and this percentage increased with subsequent CDs [18]. The difficulty of surgery was greater for our multiple caesarean sections, due to the difficulty of opening the abdominal wall, difficult separation of the vesicouterine plica, and peritoneal adhesions.

According to the above, the duration of surgery was longer in the multiple caesarean section group. The average surgical time was 62 min for our third cesareans versus 76 min for multiple cesareans (*p* < 0.001). Regarding surgical duration, the literature reports longer times for higher-order CDs [6,15,19]. However, the disparity in the variable definition may influence the quantitative comparison. 

The rate of emergency surgery was numerically lower for the multiple CD group in our study (*p* = 0.268), reflecting the clinicians’ concerns about untimely surgery. Others have described similar results [19]. It has been proposed that elective termination could be associated with a lower number of maternal complications (*p* < 0.05), such as hysterectomy, bladder injury, infectious complications, or thromboembolism in women with multiple CDs [20]. Gasim describes an urgent caesarean section rate similar to ours, with 7.6% for the group of fourth or more caesarean sections and 10.4% for the group of third caesarean sections [19]. Reviewing the literature in this regard, according to Egic, elective termination was associated with a lower number of maternal complications (12.9 versus 27.3%; *p* < 0.05), including a wide variety of complications such as hysterectomy, bladder injury, infectious complications, thromboembolism, and others [20].

We did not have cases of uterine rupture in our series, although uterine scar dehiscence was more frequent in the multiple CD group. Uterine rupture is a relatively infrequent maternal complication but potentially fatal, and its incidence has been found to be higher in women with multiple CDs [1,9,12]. Dehiscence is more common but rarely poses a serious complication [21]. Some recent works have considered both complications together. However, these complications have a different impact; therefore, collecting data on these complications independently would be desirable. Navara et al. (2020) reported an increased rate of dehiscence or rupture with a higher number of previous CDs (*p* = 0.023), which was more evident in women with ≥ 4 CDs [12]. Gasim et al. (2013), registering uterine rupture independently, described a higher incidence for the ≥ 4 CD group than for the < 4 CD group (*p* = 0.001) [19]. Placental complications are also frequent in women subjected to multiple CDs [12,15,22]. The last published work described that women with five or more previous CDs have a higher risk of placenta previa (OR = 9.8; 95% CI = 3.3–28.6) and placenta accreta (OR = 26.5; 95% CI = 4.2–166.3) [12].

In line with what has been published, no differences were found in neonatal weight, arterial pH at birth, the need for admission to the ICU, or the reason for ICU admission [6,19].

Our small sample size is clearly the reason for the disparity between our results and those reported in the literature on peripartum haemorrhage, blood transfusion, and ICU admission rates in pregnant women. It also prevents us from drawing conclusions regarding other important complications such as placenta praevia, placenta accreta, uterine dehiscence, or uterine rupture. In our case we had a pregnant woman with placenta praevia and a fourth caesarean section that ended in an obstetric hysterectomy. Several studies, including those of Miller, Silver, and more recently Narava, show an increase in placental accreta for each additional caesarean section. And so, in the latest work published in 2020, it is described that women with five or more previous cesarean sections have a 10 times higher risk of placenta previa (odds ratio [OR], 9.8; 95% confidence interval [CI], 3.3–28.6) and a 27 times increased risk of placenta accreta (OR, 26.5; 95% CI, 4.2–166.3) [12,15,22]. 

As we can see, the main limitation of our study is its small sample size. Nevertheless, due to the infrequency of these pregnancies in our environment, it is possible that this is one of the longest series of multiple caesarean sections in our country. For this reason, we believe it is relevant to present the reality of this subgroup of patients and the maternal and neonatal results found, which are on the whole very favourable. No uterine rupture or maternal-neonatal mortality was observed in any of the study groups [6,19].

Multiple studies have highlighted the risks of higher-order caesarean sections. However, the limit of “safe” caesarean sections seems to be neither clear nor sufficiently studied in our setting. For a minority group of patients considering a new gestation in the context of three or more previous caesarean sections, unification of follow-up and surgery in reference centres such as Clínica Universidad de Navarra should be considered, in order to minimise possible complications. Multicenter studies are needed in this regard in order to clarify the real risks of high-order caesarean sections in environments with high capacities, resources, and multidisciplinary teams prepared to carry out this type of complex surgery safely. For this reason, we believe it would be wise to continue this work with a prospective, multicentre study in collaboration with other centres that receive and care for this type of pregnancy, so that in an average period of time we could have a sufficient number of patients for an analysis of greater statistical power.

On the other hand, we have not analysed in this study the data relating to the experience of this type of pregnancy by patients and their families, as well as the perception of the risks transmitted by clinicians. It would be appropriate to conduct a survey to assess these items in order to know the reality of having multiple caesarean sections in our country. We have not found this type of study in the reviewed literature.

The main limitation of our study was the small sample size, which could explain the disparity between our results and those reported in the literature regarding peripartum bleeding, blood transfusion, and ICU admission rates. It also prevents drawing conclusions regarding other important complications, such as placenta previa, placenta accreta, uterine dehiscence, or uterine rupture, as we did not identify many cases in our sample. Nevertheless, these patients are infrequent in our environment; thus, the present study can be one of the most extensive series of women with multiple CDs in Spain. For this reason, our findings in this subgroup of patients, which were very favorable overall, add evidence about the maternal and neonatal outcomes in women undergoing repeat CD in our setting.

## 5. Conclusions

Although several studies have highlighted the risks of higher-order CDs, the limit for “safe” CDs seems to be unclear and poorly studied in our setting. For a minority of women considering a new gestation after three or more previous CDs, unification of follow-up and surgery in reference centers such as ours should be considered to minimize complications. Multicenter studies are needed to clarify the real risks of a high-order CD in environments with higher capacities and resources and multidisciplinary teams prepared to safely carry out this type of complex surgery. Thus, it would be wise to continue this work with a prospective, multicenter study with a larger sample to draw robust conclusions.

## Figures and Tables

**Table 1:** Sociodemographic and gestational characteristics of women in each study group.

Variables	3rd CD (*n* = 100)	Multiple CD (*n* = 61)	*p*-Value
Age, mean (SD)	35.6 (4.1)	36.0 (4.0)	0.589
BMI, mean (SD)	26.6 (3.6)	27.8 (5.1)	0.146
Obstetric pathology, N (%)			0.106
No	76 (76.0)	41 (67.2)	
Gestational Diabetes	5 (5.0)	1 (1.6)	
Thyroid pathology	5 (5.0)	12 (19.7)	
Thrombophilia	5 (5.0)	3 (4.9)	
Gestational Hypertension	1 (1.0)	0	
Fetus SGA	2 (2.0)	0	
Placenta praevia	1 (1.0)	1 (1.6)	
Multiple sclerosis	1 (1.0)	0	
Asthma	2 (2.0)	0	
Pneumonia	0	2 (3.3)	
Interpregnancy interval (months), mean (SD)	35.7 (25.3)	34.6 (18.8)	0.789

BMI: body mass index; CD: cesarean delivery; SD: standard deviation; SGA: small for gestational age.

**Table 2:** Characteristics related to surgery according to the study group.

Variables	3rd CD (*n* = 100)	Multiple CD (*n* = 61)	*p*-Value
Completion, N (%)			0.268
Scheduled	86 (86.0)	56 (91.8)	
Urgent	14 (14.0)	5 (8.2)	
Gestational age on the day of CD (days), mean (SD)	267 (7.3)	264 (6.6)	0.034 *
Type of uterine incision, N (%)			0.394
Low transverse incision	97 (97.0)	61 (100.0)	
Longitudinal incision	2 (2.0)	0	
Body incision	1 (1.0)	0	
Segment condition, N (%) ^ŧ^			0.006 *
Standard	73 (78.5)	32 (58.2)	
Very thin (<2 mm)	14 (15.1)	10 (18.2)	
Dehiscent	6 (6.5)	13 (23.6)	
Difficult abdominal opening, N (%) ^ŧ^			<0.001 *
No	47 (51.1)	10 (18.2)	
Yes	45 (48.9)	45 (82.9)	
Peritoneal adhesions ^ŧ^			0.001 *
No	88 (96.7)	43 (78.2)	
Yes	3 (3.3)	12 (21.8)	
Difficult bladder separation ^ŧ^			<0.001 *
No	59 (64.1)	15 (27.3)	
Yes	33 (35.9)	40 (72.7)	
Difficult fetal extraction			0.751
No	86 (86.0)	54 (88.5)	
Forceps	6 (6.0)	4 (6.6)	
Kiwi	8 (8.0)	3 (4.9)	
Surgical time (minutes), mean (SD)	63.0 (19.7)	76.4 (32.3)	0.001 *

CD: cesarean delivery; SD: standard deviation. * Statistically significant. ^ŧ^ There were missing cases for some variables.

**Table 3:** Obstetric hemorrhage and maternal complications according to study group.

Variables	3rd CD (*n* = 100)	Multiple CD (*n* = 61)	*p*-Value
Postpartum hemorrhage, N (%)			0.522
No	93 (93.0)	55 (90.2)	
Yes	7 (7.0)	6 (9.8)	
Severity of hemorrhage, N (%)			0.808
No	93 (93.0)	55 (90.2)	
Resolved with drugs	6 (6.0)	5 (8.2)	
Resolved with surgery or Bakri ^ŧ^	1 (1.0)	1 (1.6)	
Transfusion requirement, N (%)			0.141
No	97 (97.0)	56 (91.8)	
Yes	3 (3.0)	5 (8.2)	
Maternal admission to ICU, N (%)			0.142
No	100 (100.0)	59 (96.7)	
Yes	0	2 (3.3)	
Peripartum hysterectomy, N (%)			0.019 *
No	100 (100.0)	57 (93.4)	
Yes	0	4 (6.6)	
Bladder injury, N (%)			0.070
No	100 (100.0)	59 (96.7)	
Yes	0	2 (3.3)	
Other complications, N (%)			0.608
No	92 (92.0)	57 (93.4)	
Wound seroma	3 (3.0)	1 (1.6)	
Thrombosed hemorrhoid	0	1 (1.6)	
Endometrioma in scar	1 (1.0)	0	
Postoperative pneumonia	0	1 (1.6)	
AHT in the postpartum period	2 (2.0)	1 (1.6)	
PDPH	1 (1.0)	0	
Drug reaction	1 (1.0)	0	

AHT: arterial hypertension; CD: cesarean delivery; ICU: intensive care unit; PDPH: postdural puncture headache. ^ŧ^ Bakri intrauterine balloon. * Statistically significant.

**Table 4:** Neonatal results according to the study group.

Variables	3rd CD (*n* = 100)	Multiple CD (*n* = 61)	*p*-Value
Neonatal weight (grams), mean (SD)	3213 (423)	3163 (313)	0.436
Arterial pH, mean (SD)	7.27 (0.05)	7.26 (0.07)	0.315
Need for admission to NICU, N (%)			0.493
No	86 (86.0)	51 (82.0)	
Yes	14 (14.0)	10 (18.0)	
Reasons for admission to the NICU, N (%)			0.669
No	86 (86.0)	51 (82.0)	
Transient tachypnea	6 (6.0)	7 (11.5)	
Observation	3 (3.0)	1 (1.6)	
Hyaline membrane disease	1 (1.0)	0	
Meconium aspiration	1 (1.0)	0	
Mild respiratory distress	2 (2.0)	1 (1.6)	
Severe respiratory distress	1 (1.0)	0	
Phototherapy for jaundice	0	1 (1.6)	

CD: cesarean delivery; NICU: neonatal intensive care unit; SD: standard deviation.

## Data Availability

Data are contained within the article.
